# Tracking Hookah Bars in New York: Utilizing Yelp as a Powerful Public Health Tool

**DOI:** 10.2196/publichealth.4809

**Published:** 2015-11-20

**Authors:** Philip B Cawkwell, Lily Lee, Michael Weitzman, Scott E Sherman

**Affiliations:** ^1^ Department of Pediatrics New York University School of Medicine New York, NY United States; ^2^ Brooklyn College Brooklyn, NY United States; ^3^ College of Global Public Health New York University New York, NY United States; ^4^ Department of Environmental Health New York University School of Medicine New York, NY United States; ^5^ NYU/Abu Dhabi Public Health Research Center Abu Dhabi United Arab Emirates; ^6^ Department of Population Health New York University School of Medicine New York, NY United States; ^7^ Department of Medicine VA New York Harbor Healthcare System New York, NY United States

**Keywords:** hookah, hookah bar, Internet, public health, Yelp

## Abstract

**Background:**

While cigarette use has seen a steady decline in recent years, hookah (water pipe) use has rapidly increased in popularity. While anecdotal reports have noted a rise in hookah bars, methodological difficulties have prevented researchers from drawing definitive conclusions about the number of hookah bars in any given location. There is no publicly available database that has been shown to reliably provide this information. It is now possible to analyze Internet trends as a measure of population behavior and health-related phenomena.

**Objective:**

The objective of the study was to investigate whether Yelp can be used to accurately identify the number of hookah bars in New York State, assess the distribution and characteristics of hookah bars, and monitor temporal trends in their presence.

**Methods:**

Data were obtained from Yelp that captures a variety of parameters for every business listed in their database as of October 28, 2014, that was tagged as a “hookah bar” and operating in New York State. Two algebraic models were created: one estimated the date of opening of a hookah bar based on the first Yelp review received and the other estimated whether the bar was open or closed based on the date of the most recent Yelp review. These findings were then compared with empirical data obtained by Internet searches.

**Results:**

From 2014 onward, the date of the first Yelp review predicts the opening date of new hookah bars to within 1 month. Yelp data allow the estimate of such venues and demonstrate that new bars are not randomly distributed, but instead are clustered near colleges and in specific racial/ethnic neighborhoods. New York has seen substantially more new hookah bars in 2012-2014 compared with the number that existed prior to 2009.

**Conclusions:**

Yelp is a powerful public health tool that allows for the investigation of various trends and characteristics of hookah bars. New York is experiencing tremendous growth in hookah bars, a worrying phenomenon that necessitates further investigation.

## Introduction

### Hookah Smoking Perceived Less Dangerous Than Cigarettes

In recent years, cigarette use has had a dramatic steady decline [1-3], whereas hookah (water pipe) use has rapidly become more popular. National Adult Tobacco Survey data show that 9.8% of adults in the United States report having ever used hookah and 1.5% currently use this alternative tobacco product [4]. Among US high-school seniors, 18% report having used hookah in the past year [5]. There is growing evidence that hookah smoking may be at least as harmful as cigarette smoking [6-8]. Research has shown that the tobacco specifically used for hookah—shisha—delivers tar, nicotine, and carbon monoxide in higher doses than cigarette use [9-11]. Hookah use has also been linked to lung cancer [12], decreased pulmonary and cardiovascular function [13,14], infertility [15], and low birth weight [12]. Furthermore, secondhand hookah smoke contains the same constituents resulting from tobacco combustion, as well as additional chemicals and carcinogens from the charcoal used to heat the product [16,17], thus posing serious health risks not only to the user, but also to hookah bar employees and nonsmoking patrons [7,17,18]. Unfortunately, numerous studies worldwide consistently report that hookah smoking is perceived to be safer and less addictive than cigarettes by both public and health care providers [8,9,19-23].

In the last decade, Internet use in the United States has expanded rapidly as millions of consumers have new ways to access the Internet, from laptops to mobile phone to tablets and other devices [24].  The Internet is used by as many as 93% of high-school students and young adults [25]. The Internet also has emerged as a new resource for tracking public health trends [26]. It is now possible to analyze Internet trends as a measure of population behavior and health-related phenomena. The social media platform Twitter has been analyzed to show how users perceive and respond to emerging tobacco products [27,28]. One database, Google Trends, a publicly available resource, allows users to compare Internet search frequency over time in various regions of the world [29,30]. Researchers have used data drawn from this source to create models that accurately predict chronic disease risk factors such as alcohol consumption by measuring Web search activity [31]. This mechanism has also been used to demonstrate the increasing popularity of hookah smoking in the United States as measured by increased search volume for hookah and related terms [32].

### Social Media and Yelp

In recent years, new forms of social media and networking have also risen. Yelp is one such site in which patrons review and share information about businesses and their services, thereby allowing local businesses to publicize themselves to the public [33,34]. Based out of San Francisco, the company has set up online communities in almost every major city in the United States and worldwide. In 2014, it received an average of 135 million visitors per month and more than 67 million total reviews [35]. Yelp is ranked in the top 40 in the United States and top 150 worldwide in number of daily visitors to the website [36].

To date, no study has examined the number, distribution, and potential proliferation of hookah bars. There is no publically available database of hookah bars at either a state or federal level. Yelp has an unharnessed, but immense, potential for providing critical public health data. In this paper, we report whether data from Yelp can be used to accurately answer the following three questions: (1) What is the number of hookah bars throughout a specific geographic region and how accurately can Yelp identify the hookah bars?; (2) What are the distribution and characteristics of hookah bars (such as whether they serve alcohol or have live music)?; and (3) What are the temporal trends in hookah bars?

## Methods

### Data

Data were provided by Yelp in the form of a spreadsheet file that captured a variety of parameters for every business listed in their database as of October 28, 2014 that was tagged as a “hookah bar” and operating in the state of New York. We also obtained similar data for “wine bar” to serve as a contemporaneous control. These parameters included the business address, categorization within the Yelp database (eg, “bars,” “hookah bars,” “lounges”), business hours, and attributes such as whether alcohol is served, the ambiance, whether Wi-Fi is available, etc. In addition, every review written for each of these businesses was provided in a separate file with the reviewer’s username removed. According to Yelp’s policy, business listings are not removed after the location is determined to be closed. These businesses may feature less prominently in search results, but this did not affect the database file that we received.

### Analyses

Two simple algebraic models were created from the data provided by Yelp. The first was created to estimate the date of opening of a hookah bar based on the date of its first Yelp review. To do this, we attempted to determine the actual date of opening for each hookah bar via Internet searches. A precise opening date could be established for 46 hookah bars. Then, the actual date of opening was compared with the date of the first published Yelp review for each hookah bar. The algebraic model compared these differences on a yearly basis and used the average difference between verified date of opening and first Yelp review as the predicted date of opening for each hookah bar. Because of low review frequency in the early years of Yelp, it was not practical or accurate to use the date of the first published review as a predictor of true opening date prior to 2010.

A second algebraic model was created that predicted whether or not a hookah bar had shut down based on the date of its latest Yelp review. The Yelp website allows its users to mark locations as closed, and this information is contained within the Yelp database. We verified that all 24 of these locations were indeed closed with phone calls to the location and Internet searches. We then combed the remaining database to find whether there were other closed locations that had not been marked as such, and 7 were identified with further phone calls and Internet searches. Of the 24 that were marked closed on Yelp, 17 had not had a Yelp review written in over 1 year. Next, we computed the number of months since the last Yelp review for each hookah bar. The range was 0-65 months, the mean was 6.3 months, the median was 1 month, and the mode was 0 months (49 bars). The model considered a hookah bar as closed if it had not received a review in the past 6 months.

To determine the geographic location of hookah bars, the addresses of all hookah bars in the Yelp database were entered into Tableau Public, a freeware data visualization software program. This allowed for the generation of a heat map that examined the density of hookah bars by zip code. We present only the heat map for New York City here as it captures 121/137 (88.3%) of the hookah bars in New York State.

The reviews received by hookah bars in New York were examined in a summative fashion by examining the total number of reviews received each month from the date of the first review to October 2014. This was compared with the number of reviews received by all of the wine bars in New York, which served as a control.

## Results

### Opening Dates of Hookah Bars in New York State

**Figure 1:**
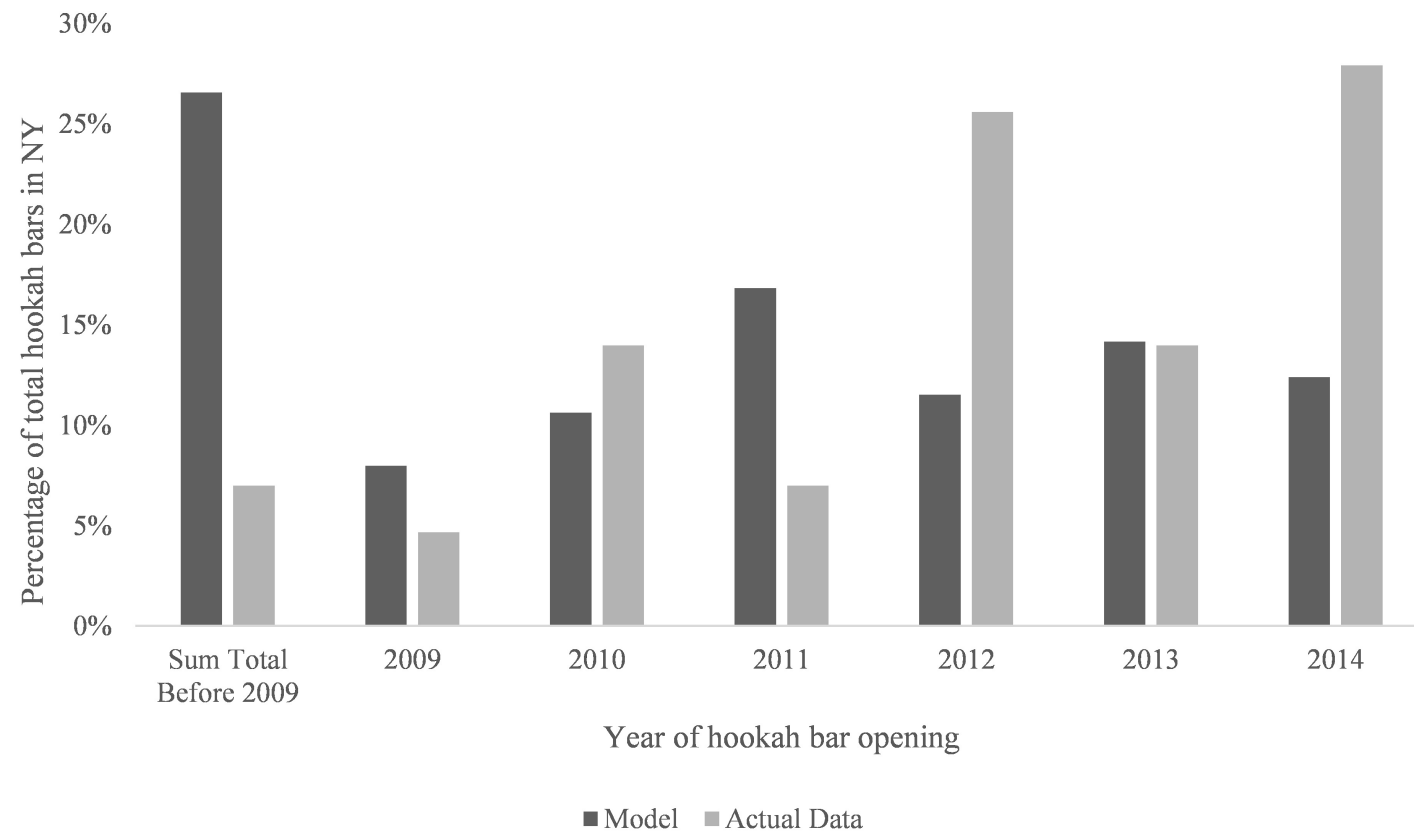
Distribution of new hookah bars by year. Actual data represents the 43 hookah bars for which a date of opening could be identified. The algebraic model uses the date of the first Yelp review for each of the 137 bars in NY (New York) to approximate its date of opening.

The Yelp database identified 137 hookah bars in the state of New York. Using a variety of aforementioned search methods, the opening date was identified for 46 currently open hookah bars. A total of 43 of the 46 opened after Yelp’s founding in 2004 and thus could be used for comparison. For bars that opened in 2010 or later, the date of the first Yelp review was a reliable proxy measure for the date of the bar opening to within 6 months (Table 1). For reviews written after January 1, 2014, only a 1-month correction factor was necessary. The date of the first Yelp review for each hookah bar was identified and this information was used to create an algebraic model that predicts the date of opening. The model predicts that 83 of the 113 (73.5%) currently open hookah bars were founded in 2009 or later, whereas the empirical data found that 40 of 43 (93%) bars opened in 2009 or later (Figure 1).

**Table 1:** Length of time between hookah bar opening and first Yelp review by year.

Real year opened	Number of hookah bars	Difference between date opening and first Yelp review (months)^a^
2006	2	34.5
2007	0	—
2008	1	22
2009	2	13
2010	6	3.5
2011	3	5.33
2012	11	3.18
2013	6	6.33
2014	12	0.75

^a^Difference calculated for the 43 hookah bars where date of opening could be verified and where the date was after Yelp’s founding (ie, after October 2004).

### Identifying Closed Hookah Bars

The Yelp database identified 24 hookah bars as closed. An algebraic model was created, using the date of the most recent Yelp review for every hookah bar in the database, to predict whether a hookah bar was open or closed. Any hookah bar that did not have a Yelp review within the last 6 months was predicted to be “closed.” A comparison of the model with the Yelp database data is provided in Tables 2 and 3. The specificity of the model is 94.7% (compared with 100% for the Yelp database “closed” tool), whereas the sensitivity of the model is 90.3% (compared with 77.4% for the raw Yelp data); the model identifies more closed bars, but makes more errors by falsely stating that bars are closed when they are open.

**Table 2:** Contingency table: Yelp-determined open/closed status of hookah bars.

	Yelp marked closed^a^	Yelp marked open
Closed hookah bars^b^	24	7
Open hookah bars	0	113

^a^“Yelp marked closed/open” refers to whether Yelp has marked the hookah bar as open or closed in their database.

^b^The closed/open hookah bar cells reflect the true status based on telephone calls and Internet searches.

**Table 3:** Contingency table: Model determined open/closed status of hookah bars.

	Model marked closed^a^	Model marked open
Closed hookah bars	28	3
Open hookah bars	6	107

^a^The algebraic model uses the date of the last review for the hookah bar to estimate whether it is open or closed. Bars that have not received a review within the last 6 months are estimated to be closed.

### Hookah Bar Locations

**Figure 2:**
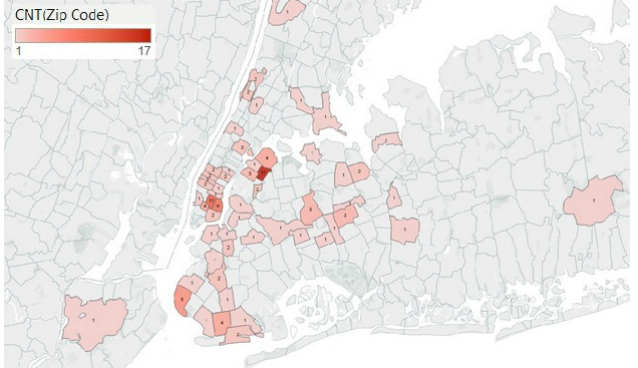
Heat map of location of hookah bars in New York City by zip code.

The location and distribution of the hookah bars were also examined. An overwhelming majority (121/137) of the hookah bars in the state of New York are located in one of New York City’s 5 boroughs. The majority are clustered in Queens (n=43), Manhattan (n=43), and Brooklyn (n=26). Figure 2 shows a heat map with the locations of hookah bars by zip code. The greatest cluster occurs in zip code 11103, a subdivision of Astoria, Queens, a 0.71-mi^2^ area [37] with 17 hookah bars. The zip codes 10002, 10003, and 10009 comprise Manhattan’s Lower East Side and 10001-10006 comprise Astoria, Queens, according to the United Hospital Fund classification [38]. These are the 2 neighborhoods in New York with the highest density of hookah bars.

Unique characteristics of the hookah bars were examined (see Multimedia Appendix 1). The information provided ranges from whether the bars accept credit cards (115/137, 83.9% yes) to whether patrons describe the ambience as “trendy” (20/137, 14.6% yes).

Finally, temporal trends were evaluated by analyzing the individual reviews received by each hookah bar. The first review of a New York hookah bar occurred on August 20, 2005; the number of hookah bar reviews has grown manifold since then. From August 2005 to August 2008, there were an average of 7.2 reviews of hookah bars per month. From August 2013 to 2014, this number jumped to 118.8. We compared the cumulative number of reviews for hookah bars with those for wine bars to control for the growth rate of Yelp. From October 2008 to 2014, the total number of hookah bar reviews increased 17-fold, compared to a 10-fold increase for wine bars.

## Discussion

### Principal Findings

In this study, we used a dataset provided by Yelp to create a simple algebraic model that identifies when a hookah bar was founded based on the date of its first review. The model estimates that there have been more new hookah bars from 2012 to 2014 than the number that existed prior to 2009. While others have anecdotally noted the evidence of rapid expansion of hookah bars [39], we have taken this a step further by identifying the year-by-year expansion seen in New York. This suggests that retailers in the form of new hookah bars have met the increase in demand for hookah. Salloum et al [32] used the novel mechanism of Internet search queries to demonstrate the expanding popularity of hookah use. The rapidly increasing frequency of reviews for hookah bars relative to wine bars on Yelp shown here seems to corroborate this. Others have conducted more traditional surveys and analyses that agree with the notion that there is high demand for hookah [5,40,41].

We also used the Yelp data to map the locations of hookah bars in New York, and specifically focused on the distinct clusters in New York City because this was where the vast majority of hookah bars were located. One cluster was Astoria, Queens, which contained 27 hookah bars, and another was the Lower East Side, which contained 22. This finding is thought provoking because a large population of college students reside in this area and a large population of immigrants [42], especially Arabs, reside in Astoria [43]. This is significant because these populations typically have high rates of tobacco use [44,45]. Others have corroborated the idea that hookah bar expansion tends to occur near colleges [46] and to attract adolescents [47]. It is not entirely surprising that business owners would want to set up shop in areas with high demand.

### Limitations

There are several limitations to this study. The first is that the precision of our algebraic models was limited by the temporally skewed data. The earliest verified date of opening of a hookah bar in New York City in our database is 1977 (the hookah component was not added until the 2000s); Yelp, however, was founded in October 2004, and this specific bar did not appear until January 2006. Thus, the model could only say that a bar was likely to have been founded “pre-2006.” It is inherently more accurate with newer bars and there is a higher level of uncertainty about the opening date of bars founded before 2009, for which the model could not accurately identify the opening date to within a year. In addition, 38 of the 46 hookah bars that we could identify had an official opening date that occurred in 2010 or later. It is possible that there is some form of recency effect occurring, as we are more likely to be able to identify more newly opened hookah bars that may have promoted themselves via the Internet. This is reflected in Figure 1, as few bars were identified with an opening date prior to 2009 and yet the model predicted many bars to have opened in that range based on their first date of Yelp review. Yelp’s expanding popularity ensured that a hookah bar was likely to have a review posted on Yelp within the first 6 months of its opening from 2010 to 2013, and within a month from 2014 onward, which results in higher confidence with the more recent data. Our comparison of hookah bar review frequency with wine bar frequency is a relative control, as it is unclear whether they have been increasing or decreasing in popularity. Thus, this comparison can only serve to provide some context for the rate of increase in hookah bar reviews. Next, our algebraic model was intentionally designed to be simplistic. This allows for a robust and rapid estimation of whether a hookah bar is open or closed and when it was founded based simply on the date of its first and most recent reviews. As a drawback, however, it is not as precise as could potentially be achieved with more comprehensive measures.

Yelp is a powerful tool and is getting still more popular; we noted a 20-fold increase in monthly reviews for hookah bars between January 2007 and 2014. In spite of this, there have been very few public health studies that utilize its vast database, and only one that used it in the context of hookah. Primack et al [48] utilized Yelp to locate hookah bars in municipalities with clean air laws. Sussman et al [34] coded Yelp reviews in Los Angeles, California, for Vape shops to analyze consumer beliefs and behaviors. Other medical studies that use Yelp data focus on analyzing reviews of physicians who are listed on Yelp [49-51] and on food safety [52,53].

Yelp offers both public health researchers and potentially local health departments the ability to quickly and accurately spot trends. We found that on average, beginning in 2014, a new hookah bar will appear on Yelp within 1 month of its opening. This allows for rapid identification of an expanding market. This could be particularly useful for local health departments. In New York City, for example, the health department is very active in enforcing health standards; they recently undertook an operation to identify which hookah bars were serving shisha-containing tobacco, which is a violation of the 2002 Smoke-Free Air Act [54]. Utilization of Yelp data would be beneficial because it would allow health departments to rapidly identify new bars. The same would apply to researchers seeking to measure the effects on working in a hookah bar or track their expanding popularity.

Some data contained within the Yelp database are more immediately useful than others are. It is readily apparent how knowledge of location of the hookah bars could be useful to researchers, health departments, and policy makers. However, there is a trove of other data collected for every hookah bar which includes whether they have parking, television, live music, offer outdoor seating, accept credit cards, or if they serve alcohol. Whether there are important findings or implications contained in these data remain to be seen, but it is clear that Yelp is an important, but underutilized, public health tool.

### Conclusions

In summary, the findings presented in this paper corroborate that data from Yelp do in fact accurately identify the supply of hookah bars and their distribution and characteristics, as well as allowing for monitoring changes in their presence over time. In the state of New York, these data demonstrate both an increasing number of such venues and substantial geographic clustering. As emerging health epidemics like hookah use grow, public health officials and researchers would be well served to consider innovative sources such as Yelp for data analysis.

## Appendix

Multimedia Appendix 1 Attributes of the 137 hookah bars in New York, as described by Yelp.
