# Supplementary material for: Tracking Hookah Bars in New York: Utilizing Yelp as a Powerful Public Health Tool
Source: JMIR Public Health Surveill. 2015 Nov 20;1(2):e19. doi: 10.2196/publichealth.4809 (PMC4869217; doi:10.2196/publichealth.4809)
Supplement: Multimedia Appendix 1 [file publichealth_v1i2e19_app1.pdf]

Multimedia Appendix 1. Attributes of the 137 hookah bars in New York, as described by Yelp.

Attribute	True	False	Blank
"Good for Groups"	94.2%	2.9%	2.9%
Accepts Credit Cars	83.9%	11.0%	5.1%
Has TV	62.0%	27.7%	10.2%
Street Parking	61.3%	27.7%	11.0%
Happy Hour	60.6%	25.6%	13.9%
Has a DJ?	54.0%	24.8%	21.2%
"Good for Dancing"	46.0%	43.1%	11.0%
Parking Lot	4.4%	84.7%	11.0%
"Trendy" Ambience	14.6%	72.3%	13.1%
No Alcohol	23.4%	68.6%	8.0%
Has TV	27.7%	62.0%	10.2%
Coat Check	23.4%	61.3%	15.3%
Outdoor Seating	33.6%	60.6%	5.8%
Has Live Music	5.1%	55.5%	39.4%
"Casual" Ambience	33.6%	53.3%	13.1%
Caters	14.6%	20.4%	65.0%
Waiter Service	44.5%	0.7%	54.7%
Free Wifi	29.9%	15.3%	54.7%
Wheelchair Accessible	32.9%	12.4%	54.7%
"Good for Late-night"	26.3%	19.7%	54.0%
Take Out	26.3%	20.4%	53.3%
Delivers	13.9%	32.9%	53.3%
Takes Reservations	32.9%	16.8%	50.4%
"Good for Kids"	4.4%	46.7%	48.9%
